# Safety and immunogenicity of heterologous recombinant protein subunit vaccine (ZF2001) booster against COVID-19 at 3–9-month intervals following two-dose inactivated vaccine (CoronaVac)

**DOI:** 10.3389/fimmu.2022.1017590

**Published:** 2022-11-08

**Authors:** Yuting Liao, Yingping Chen, Bo Chen, Zhenzhen Liang, Xiaosong Hu, Bo Xing, Juan Yang, Qianhui Zheng, Qianhui Hua, Chuanfu Yan, Huakun Lv

**Affiliations:** ^1^ State Key Laboratory of Molecular Vaccinology and Molecular Diagnostics, National Institute of Diagnostics and Vaccine Development in Infectious Diseases, School of Public Health, Xiamen University, Xiamen, Fujian, China; ^2^ Department of Immunization Program, Zhejiang Provincial Center for Disease Control and Prevention, Hangzhou, Zhejiang, China; ^3^ Kaihua Center for Disease Control and Prevention, Quzhou, Zhejiang, China; ^4^ School of Medicine, Ningbo University, Ningbo, Zhejiang, China

**Keywords:** COVID-19, heterologous booster, recombinant protein subunit vaccine, inactivated vaccine, immunogenicity, safety

## Abstract

**Background:**

In response to SARS-CoV-2 mutations and waning antibody levels after two-dose inactivated vaccines, we assessed whether a third dose of recombinant protein subunit vaccine (ZF2001) boosts immune responses.

**Methods:**

An open-label single-center non-random trial was conducted on people aged 18 years and above at five sites in China. All participants received a two-dose inactivated vaccine (CoronaVac) as their prime doses within 3–9 months of the trial. Primary outcomes were safety and immunogenicity, primarily the geometric mean titers (GMTs) of neutralizing antibodies to live wildtype SARS-CoV-2.

**Results:**

A total of 480 participants (median age, 51; range 21–84 years) previously vaccinated with two-dose CoronaVac received a third booster dose of ZF2001 3–4, 5–6, or 7–9-months later. The overall incidence of adverse reactions within 30 days after vaccination was 5.83% (28/480). No serious adverse reactions were reported after the third dose of ZF2001. GMTs in the 3–4-, 5–6-, and 7–9-month groups before vaccination were 3.96, 4.60, and 3.78, respectively. On Day 14, GMTs increased to 33.06, 47.51, and 44.12, respectively. After the booster, GMTs showed no significant difference among the three prime-boost interval groups (all P>0.05). Additionally, GMTs in older adults were lower than those in younger adults on Day 14 for the three groups (P=0.0005, P<0.0001, and P<0.0001).

**Conclusion:**

Heterologous boosting with ZF2001 was safe and immunogenic, and prime-boost intervals did not affect the immune response. The immune response was weaker in older than younger adults.

## Introduction

The COVID-19 pandemic caused by severe acute respiratory syndrome coronavirus 2(SARS-CoV-2) is ongoing worldwide (1). According to the Johns Hopkins Coronavirus Resource Center, more than 572 million people have been infected with COVID-19, which has caused 63 million deaths worldwide as of July 27, 2022 (2). Vaccination with a supply of multiple vaccines aimed to control pandemic is based on different platforms (3). In previous studies, vaccines have shown remarkable effectiveness as protection against COVID-19 infection and especially severe disease cases and death. CoronaVac inactivated vaccine was found to effectively prevent COVID-19, including severe disease and related deaths. The vaccine’s effectiveness in preventing COVID-19 in phase 3 clinical trials was 83.5%, 65.9%, and 50.38% in Turkey, Chile, and Brazil, respectively (4–6). The results of a clinical study showed the efficacy of the ZF2001 recombinant protein subunit vaccine to be 75.7% for symptomatic COVID-19 cases, 87.6% for severe-to-critical COVID-19 cases, and 86.5% for COVID-19-related deaths (7).

Currently, highly transmissible SARS-CoV-2 variants of concern (VOC), such as the Omicron SARS-CoV-2 variant, have led to new waves of infection globally, despite the increase in the number of people who are vaccinated. The immunity generated by vaccines and previous infections is less effective against the predominant strain Omicron, which accounts for many breakthrough infections. Owing to its reduced sensitivity to currently available vaccines, Omicron represents a more challenge to vaccine efficacy (8). Omicron neutralization data from 23 laboratories showed the antibody titer to Omicron decreased more than 19-fold relative to that for the wild type in twice-vaccinated individuals (9). Recently, the protective effectiveness of COVID-19 vaccines has been in decline over time due to fading humoral immunity. There is evidence that humoral immune responses decline quickly within 6 months after a standard two-dose regimen of inactivated SARS-CoV-2 vaccination (10). A recent study showed that, regardless of age, gender, or chronic diseases of recipients, total antibody levels begin to decline from 6 weeks after full BNT162b2 vaccination and drop by more than 50% within 10 weeks (11).

In response to the mutations of COVID-19 variants and waning antibody levels, strategies to improve antibody levels to COVID-19 have been implemented in many countries. Previous research demonstrated that a third dose of CoronaVac administered to adults 8 months after the first two doses effectively reinstated specific immune responses to SARS-CoV-2 (12). Additionally, a third booster dose of inactivated vaccine provided a better breadth of VOC neutralization ability, expeditious immune recall, and a long-lasting humoral response to SARS-CoV-2 (13). The use of BNT162b2 and mRNA-1273 as homologous third booster doses administered 6–8 months after the second dose also greatly increased neutralizing antibody concentrations. Compared with a two-dose schedule, a third booster dose provided substantially increased protection against symptomatic infection with the Omicron variant (14). To promote greater protection, heterologous prime-boost vaccination schedules have been explored based on their successful use in clinical trials and real-world studies. Several clinical trials exploring heterologous vaccination schedules with the combinations ChAdOx1 nCoV-19 and BNT162b2, Ad26.COV2-S and BNT162b2, BNT162b2 and Ad26.COV2-S, CoronaVac and ChAdOx1 nCoV-19, and CoronaVac and Convidecia (15–18), demonstrated that a heterologous prime-boost vaccination schedule can be more immunogenic than a homologous schedule in adults aged 18 years and older. These results indicated the flexibility possible in the deployment of COVID-19 vaccines and the benefits of mix and match schedules.

A recent heterologous vaccination strategy implemented in China involves the administration of a recombinant protein subunit vaccine (ZF2001) as a third dose after a full schedule of a two-dose inactivated vaccine. In this study, given the urgent need for data to inform policy making, we evaluated the safety and immunogenicity of administering a recombinant protein subunit booster vaccine (ZF2001) against COVID-19 following a two-dose inactivated vaccine (CoronaVac) in adults aged 18 years and older.

## Methods and materials

### Study design and participants

This study was designed as a single-center open-label trial to access the safety and immunogenicity of a recombinant protein subunit booster vaccine (ZF2001) against COVID-19 following a two-dose inactivated vaccine (CoronaVac). The study was conducted at five study sites(Majin, Chihuai, Huabu, Qinyang, Yinken) in community health centers in Kaihua County, Zhejiang Province, China, by the Zhejiang Provincial Center for Disease Control and Prevention (CDC). Eligible participants were initially recruited and allocated (1:1:1) to various prime-boost interval groups: a 3–4-month group (Group A), a 5–6-month group (Group B), and a 7–9-month group (Group C). For every group, participants aged 18–59 years and ≥60 years were assigned at a ratio of 3:1.

All participants were required to have no or well-controlled comorbidities. Key exclusion criteria were previous SARS-CoV-2 infection, history of anaphylaxis, history of allergy to a vaccine ingredient, pregnancy, breastfeeding, intent to conceive, and current use of anticoagulants. Full details of the inclusion and exclusion criteria can be found in the **Supplementary Methods**.

Ethics approval was obtained from the Zhejiang Provincial Center for Disease Control and Prevention (2021–029–01) for this study. Signed informed consent from each participant was obtained before screening, and the trial was conducted in accordance with the Helsinki Declaration, Good Clinical Practice, and Chinese regulatory requirements.

### Study vaccine

The recombinant protein subunit COVID-19 vaccine, ZF2001, was jointly developed by the Institute of Microbiology, Chinese Academy of Sciences, and Anhui Zhifei Longcom Biopharmaceuticals and manufactured by Anhui Zhifei Longcom Biopharmaceuticals (19). The vaccine uses the tandem-repeat dimeric receptor-binding domain (RBD) of the SARS-CoV-2 spike protein (from the original Wuhan-Hu-1 strain) as the antigen. This vaccine was manufactured in the CHOZN CHO K1 cell line and produced as a liquid formulation containing 25 μg of NCP-RBD protein per 0.5 mL in a vial, with aluminum hydroxide as the adjuvant. All participants received the vaccine intramuscularly in their upper arms. In this trial, the batch number of the vaccine was A202107150.

### Data collection

After vaccination, all participants were monitored for 30 min for immediate adverse reactions and were trained to record all adverse events occurring within 7 days after injection on diary cards. Adverse events occurring 8–30 days later were recorded in contact cards. Throughout the trial, participants were requested to record all serious adverse events and pregnancy outcomes. Adverse events were graded according to the National Medical Products Administration of China (2019 version). The causal association between vaccination and adverse events was determined by the trained investigators.

Serum samples were collected to evaluate neutralizing activity against live wildtype SARS-CoV-2 before vaccination (Day 0) and on Day 14 post-vaccination for all participants. Neutralizing activity was assessed against live wildtype SARS-CoV-2 by cytopathic effect (CPE) assay.

### Live SARS-CoV-2 neutralization assay

Quantifying neutralizing antibodies to live SARS-CoV-2, CPE assay was used to determine the 50% neutralization titer. Briefly, a portion of serum was diluted with an equal volume of DMEM, incubated at 56°C for 30 min for safety, and then diluted 2-fold in 96-well plates serially. All samples were incubated for 1 h at 37°C with 50% tissue culture infectious dose (TCID50) of SARS-CoV-2. Next, the serum/virus mixtures were added to VeroE6 cells in another 96-well plate and incubated at 37°C for 1 h. After a 3-day incubation, the plate was observed under a microscope and the CPE of each well was recorded. The neutralizing titers were set as the absence of 50% CPE compared with virus controls, and calculated with the Reed–Muench or Spearman–Kärber method. The positive cutoff for defining seropositivity for neutralizing antibodies to live SARS-CoV-2 was 1:4, and neutralizing antibody detection data below the cutoff value were as 1:2 included in the immunology analysis.

### Outcomes

The co-primary outcomes were the safety and immunogenicity of the ZF2001 booster following two-dose CoronaVac. Safety outcomes were the occurrence of adverse events following vaccination, including all adverse events, adverse events related to vaccination, adverse events of grade 3 and worse, and adverse events leading to withdrawal of participants. All adverse events and serious adverse events related to vaccination in all groups were analyzed.

The immunogenicity outcome was defined as neutralizing antibody titers against the wild type (WT; the original strain identified in Wuhan) on day 14 of follow-up. The primary immunogenicity endpoints of neutralizing titers in each group were reported as geometric mean titers (GMTs) at pre-immunization and Day 14 after booster vaccination. Second immunogenicity endpoints included the geometric mean ratio (GMR) of neutralizing titers among each subgroup, seroconversion rates, and positive rates on Day 14. The seroconversion rate was defined as a change in neutralizing titers from seronegative at baseline to seropositive, or a four-fold increase in titers for participants whose neutralizing titers were above the seropositive cutoff (1:4). Positive rates were defined as the proportion of people whose neutralizing titers were 1:4 and above on Day 14 after vaccination.

### Sample size calculation

The sample size of each group was calculated according to single-arm objective performance criteria. According to the immunogenicity data of the recombinant novel coronavirus vaccine (CHO) phase II clinical trial, the GMT of subjects over 18 years old after three doses was 102.5, the expected GMT was 105.5, and the standard deviation was 10.5, with α = 0.025 (one-sided) and β = 0.10. Considering 20% loss to follow-up, the sample size was 132 people in each group, which was adjusted to 160 people. According to the prime-boost interval, three groups were set up in this study, so the total sample size was 480 people. Among them, Z_1_-_α_ and Z_1_-_β_ represented the corresponding quantiles of 1-α and 1-β in the standard normal distribution, μ_1_ was the expected GMT, μ_0_ was the target GMT, and σ was the standard deviation.


$$n=\frac{{\left(Z_{1-\alpha }+Z_{1-\beta }\right)}^{2}\sigma^{2}}{{\left(\mu_{1}-\mu_{0}\right)}^{2}}$$

### Statistical analysis of data

Primary analysis for immunogenicity outcomes was per-protocol set (PPS) and included all participants who had no severe protocol violations and completed two blood collections. Safety assessments were done on a safety population dataset of all participants who received a third dose.

The characteristics of participants in the three prime-boost interval groups (Group A-C) were summarized. Pearson χ^2^ test and Fisher’s exact test were used to analyze categorical data. GMTs were calculated from the log2-transformed neutralizing antibody titers. GMRs were calculated from the difference between the mean of the log2-transformed neutralizing antibody titers in the different two groups. Comparisons of GMTs between baseline and post-vaccination in individuals were conducted with paired t tests or Wilcoxon signed-rank tests depending on the normality of the log2-transformed neutralizing antibody titer data. Comparisons between 18–59 and ≥60-year-old participants within groups on Day 14 were analyzed with t tests or Wilcoxon rank sum tests. Comparisons by age subgroup were analyzed with ANOVA or Kruskal–Wallis H test. The number and proportion of participants with adverse events after vaccination were described. Correlation analysis between age and fold change were assessed by the Spearman correlation coefficient. Significance was set at *P*< 0.05 (two-sided). We used Microsoft Excel 2019, SPSS 24.0 statistical software, and GraphPad Prism 9 to analyze data and generate graphs.

## Results

### Participants

**Figure 1:**
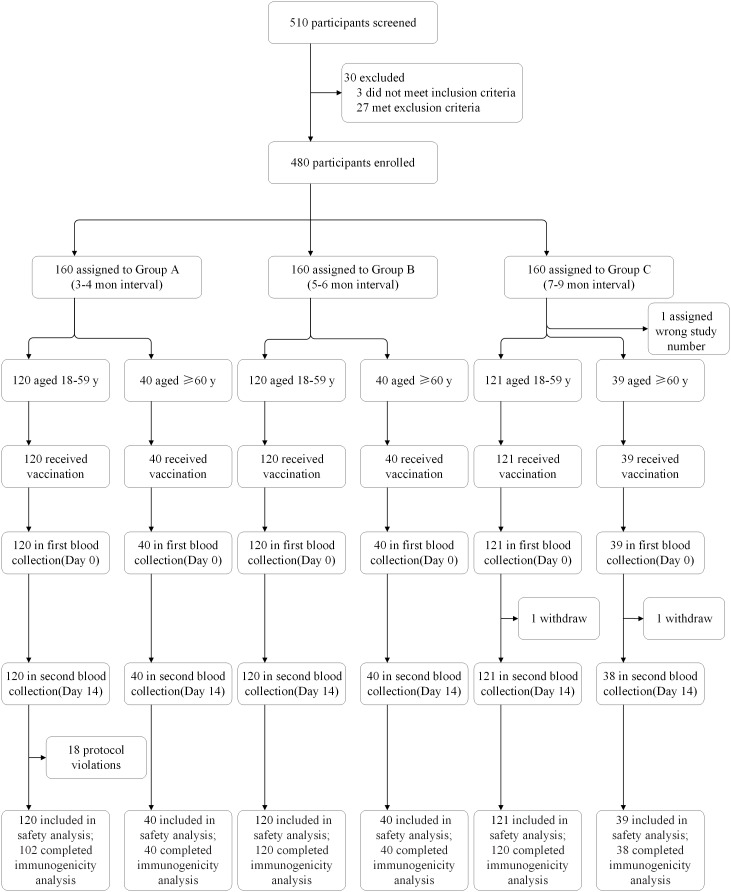
Trial profile. Protocol violations: participants who did not receive two doses of CoronaVac, but other inactivated vaccines, were included from the safety analysis.

Between October 21 and December 6, 2021, 510 people were screened, of whom 480 were enrolled (**Figure 1**). In each of the three groups vaccinated at different time intervals (Group A-C), 160 participants were divided into two age subgroups of 120 participants aged 18–59 years and 40 participants aged 60 years or more. All enrolled participants were included in the safety population. There were eighteen participants aged 18–59 years in Group A who had protocol violation and two participants in Group C who withdrew before blood collection on Day 14 excluding from the per-protocol population for immunology evaluation.

The enrolled participants had a median age of 51 years (range 21–84). The median age of participants in Group A was 51.1 years (range 25–78), including a median age of 49 years (25–59) in the younger age group and 64 years (61–78) in the older age group (**Table 1**). Group B participants had a median age of 51 years (26–84), including median age of 48 years (26–59) in the younger age group and 68.5 years (60–84) in the older age group. Participants in Group C had a median age of 51 years (21–84), including a median age of 48 years (21–59) in the younger age group and 68 years (60–84) in the older age group. Of the 480 participants, 252 (52.5%) were female and 228 (47.5%) were male. In groups A, B and C, 86 (53.7%), 91 (56.9%), and 75 (46.9%) participants were female, while 74 (46.3%), 69 (43.1%), and 85 (53.1%) participants were male, respectively. Almost all (99.6%) participants were Han Chinese, except for 2 (0.4%) participants of other races in group A.

**Table 1:** Baseline characteristics of enrolled participants.

	Group A (N=160)	Group B (N=160)	Group C (N=160)	Overall (N=480)
Age (years)				
Mean (SD)	52.0 (10.7)	51.8 (12.5)	51.9 (13.2)	51.9 (12.2)
Median (range)	51.5 (25-78)	51.0 (26-84)	51.0 (21-84)	51.0 (21-84)
18-59y (N)	120	120	121	361
Mean (SD)	47.5 (8.1)	46.2 (8.4)	46.2 (9.3)	46.6 (8.6)
Median (range)	49.0 (25-59)	48.0 (26-59)	48.0 (21-60)	48.0 (21-60)
≥60y (N)	40	40	39	119
Mean (SD)	65.6 (4.1)	68.5 (6.1)	69.6 (6.1)	67.9 (5.7)
Median (range)	64.0 (61-78)	68.5 (60-84)	68.0 (60-84)	67.0 (60-84)
Sex				
Male n (%)	74 (46.3)	69 (43.1)	85 (53.1)	228 (47.5)
Female n (%)	86 (53.7)	91 (56.9)	75 (46.9)	252 (52.5)
Race				
Han population n (%)	158 (98.8)	160 (100.0)	160 (100.0)	478 (99.6)
Others n (%)	2 (1.2)	0 (0.0)	0 (0.0)	2 (0.4)
Comorbidities				
Yes n (%)	45 (28.1)	44 (27.5)	52 (32.5)	141 (29.4)
No n (%)	115 (71.9)	116 (72.5)	108 (67.5)	339 (70.6)
Medication history				
Yes n (%)	32 (20.0)	35 (21.9)	43 (26.9)	110 (22.9)
No n (%)	128 (80.0)	125 (78.1)	117 (73.1)	370 (77.1)
Vaccination within 28 days				
Yes n (%)	2 (1.3)	0 (0.0)	0 (0.0)	2 (0.4)
No n (%)	158 (98.7)	160 (100.0)	160 (100.0)	478 (99.6)

### Safety

**Figure 2:**
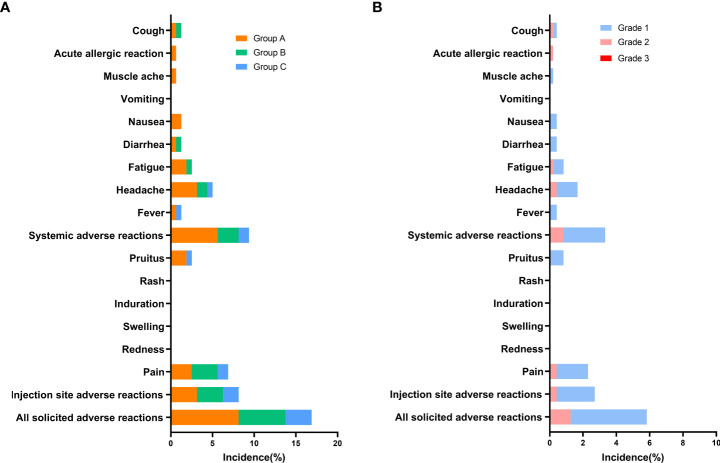
Incidence of solicited adverse reactions reported within 30 days after booster dose of ZF2001 (safety analysis population). Adverse reactions refer to adverse events related to the vaccination. **(A)** Incidence of solicited adverse reactions in groups of different prime-boost time intervals (Group A, B, C). **(B)** Incidence of solicited adverse reactions of different grades (Grade 1, 2, and 3). Unsolicited adverse reactions are not shown in the figure.

The overall incidence of adverse reactions within 30 days after vaccination was 5.83% (28/480), and they were predominantly solicited adverse reactions (5.63%, 27/480) (**Table S1**). Pain (2.29%) was the most common solicited injection-site adverse reaction, and headache (1.67%) and fatigue (0.83%) were the most common solicited systemic reactions (**Figure 2**). Pruitus (0.83%), fever (0.42%), diarrhea (0.42%), nausea (0.42%), and cough (0.42%) also occurred after vaccination. The incidences of any solicited adverse reactions were similar among Groups A–C, at 8.13%, 5.63%, and 3.13%, respectively. Dizziness (0.21%) was the only unsolicited adverse reaction. For all adverse reactions, there were grade 1 (4.79%) and grade 2 (1.25%) adverse reactions. Of the solicited injection-site adverse reactions, grade 1 and 2 adverse reactions accounted for 2.29% and 0.42%, respectively. Of the solicited systemic adverse reactions, grade 1 and 2 reactions accounted for2.5% and 0.83%, respectively. All unsolicited adverse reactions were grade 1 (0.21%). There were no adverse reactions of grade 3 occurring with 30 days.

### Immunogenicity

**Figure 3:**
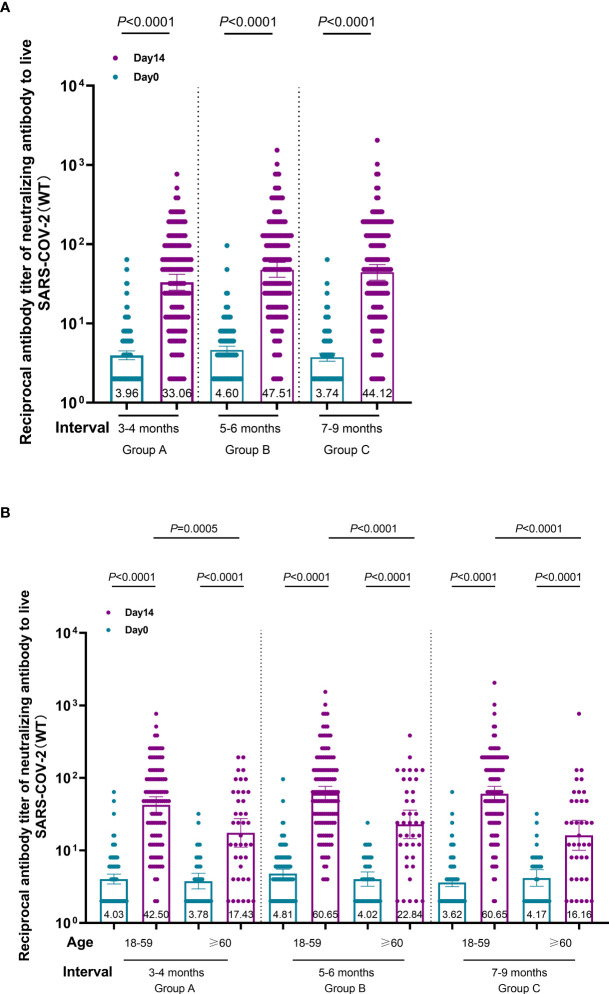
Immunogenicity of the booster vaccine (ZF2001) in participants of each group on Day 0 and Day 14. **(A)** Titers of the different prime-boost time interval groups measured by infectious SARS-CoV-2 neutralizing assay. Circles show the individual neutralization titers. Bars represent the geometric mean titers (GMT) of neutralization antibody. Error bars refer to the 95% CI. Negative in neutralization antibody detection is represented as GMT = 2. **(B)** Titers of the different time interval groups and age subgroups measured by infectious SARS-CoV-2 neutralizing assay. The GMTs of participants aged 18–59 years and ≥60 years were compared among the three groups on Day-14 post-vaccination. Circles show the individual GMTs. Error bars refer to the 95% CI.

**Figure 4:**
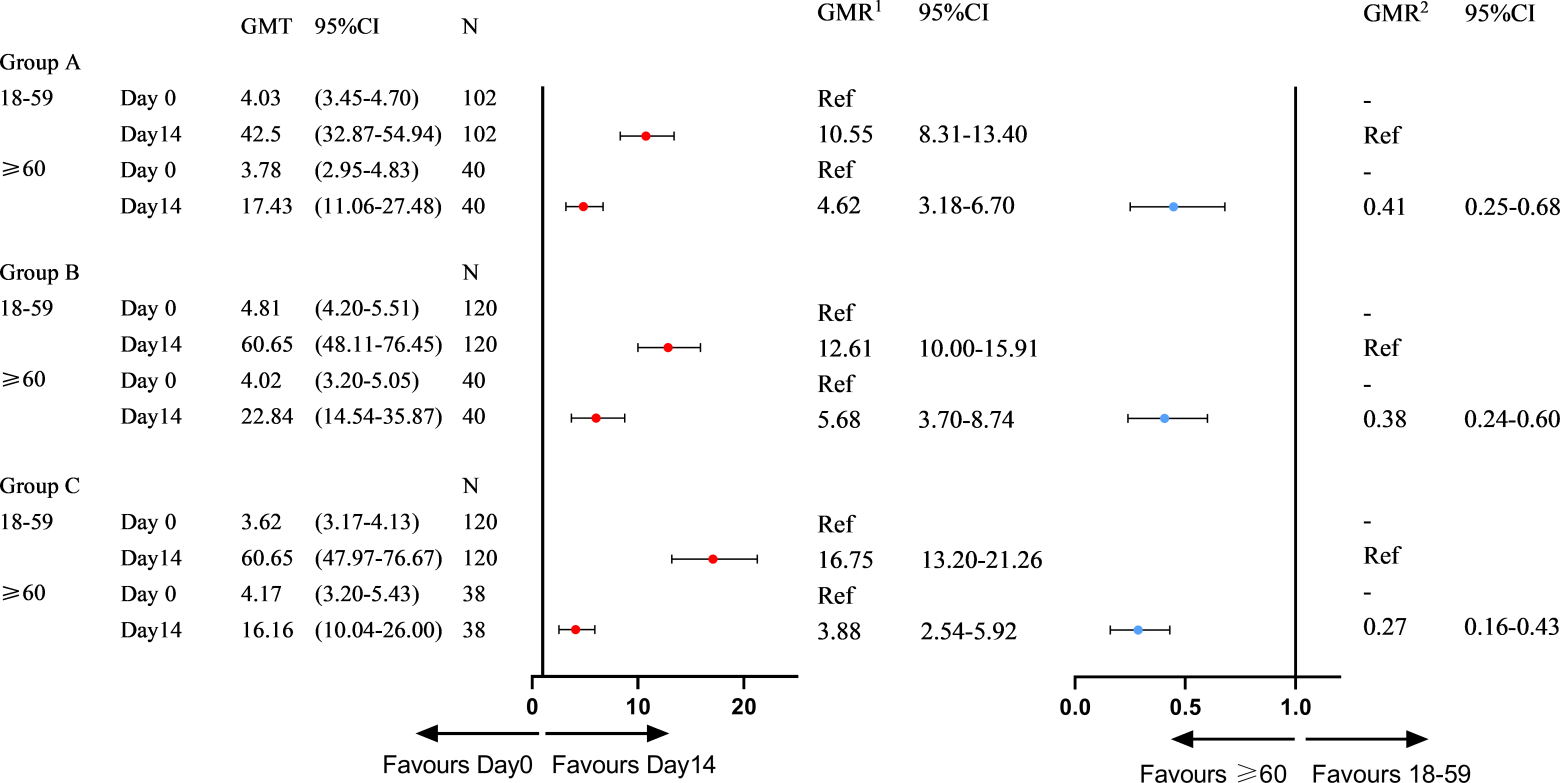
Subgroup immunogenicity analysis by age on Day 0 and Day 14 for three prime-boost intervals. GMT, geometric mean titer; N, number of per-protocol set in immunogenicity analysis; GMR^1^, geometric mean ratio on Day 14 vs Day 0 in different age subgroups; GMR^2^, geometric mean ratio of ≥60 vs 18-59 years of age on Day 14; CI, confidence interval.

**Figure 5:**
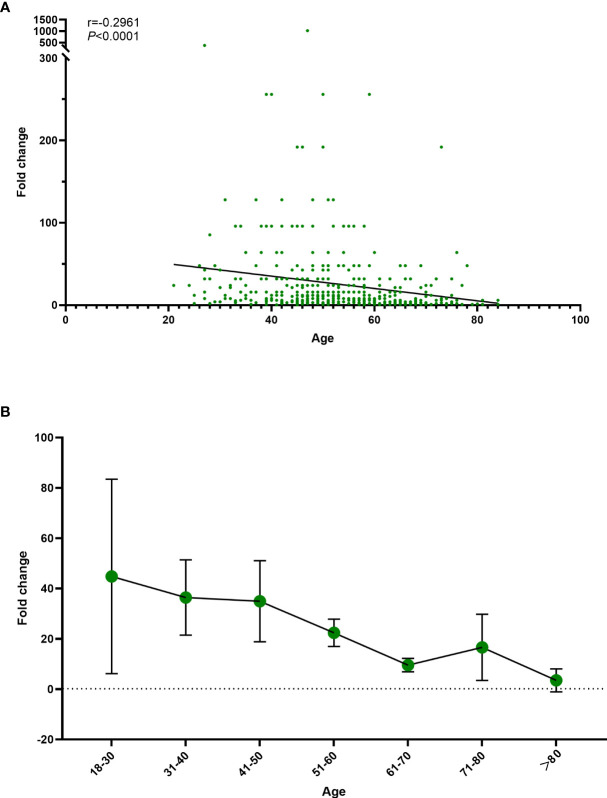
Association of age with the fold change in neutralizing antibody titers. **(A)** Correlation between the fold change in neutralizing antibody titers and age. **(B)** Trend in the fold change in neutralizing antibody titers with age. Fold change= Ratio of neutralizing antibodies before and after vaccination. r; Spearmen correlation coefficient.

At the 3–9-month interval after primary immunization with CoronaVac, neutralizing antibody levels against SARS-CoV-2 (WT) were close to the negative detection point (titer<1:4) for half of the individuals. The third dose of ZF2001 vaccine rapidly induced a significantly high degree of humoral immunogenicity (all *P*< 0.0001) (**Figure 3A**). For participants in the 3-4-month (A), 5–6-month (B), and 7–9-month (C) interval groups, the GMTs of neutralizing antibodies to live SARS-CoV-2 (WT) on Day 0 before vaccination were 3.96, 4.60, and 3.78, respectively. On Day 14, the respective GMTs of neutralizing antibody increased to 33.06, 47.51, and 44.12. For participants aged 18–59 and ≥60 years in Group A, the respective GMTs (95%CI) of neutralizing antibodies to live SARS-CoV-2 (WT) on Day 0 before vaccination were 4.03 (95%CI: 3.45–4.70) and 3.78 (95%CI: 2.95–4.83), which increased significantly to 42.50 (95%CI: 32.87–54.94) and 17.43 (95%CI: 11.06–27.48) after ZF2001 vaccination, with GMRs of 10.55 (95%CI: 8.31–13.40) and 4.62 (95%CI: 3.18–6.70), respectively (**Figures 3B**, **4**). Similarly, there was a significant increase in GMT from Day 0, 4.81 (95%CI: 4.20–5.51) and 4.02 (95%CI: 3.20–5.05), to Day 14, 60.65 (95%CI: 48.11–76.45) and 22.84 (95%CI: 14.54–35.87), with GMRs of 12.61 (95%CI: 10.00–15.91) and 5.68 (95%CI: 3.70–8.74) for participants aged 18–59 years and ≥60 years, respectively, in Group B. GMTs also increased considerably from Day 0, 3.62 (95%CI: 3.17–4.13) and 4.17 (95%CI: 3.20–5.43), to Day 14, 60.65 (95%CI: 47.97–76.67) and 16.16 (95%CI: 10.04–26.00), with GMRs of 16.75 (95%CI: 13.20–21.26) and 3.88 (95%CI: 2.54–5.92) for participants aged 18–59 and ≥60 years, respectively in Group C. Additionally, the GMTs in the older age group were lower than those in the younger age group on Day 14, with GMRs of 0.41 (95%CI: 0.25–0.68), 0.38 (95%CI: 0.24–0.60), and 0.27 (95%CI: 0.16–0.43) in Groups A–C (*P* = 0.0005, *P<* 0.0001, and *P<* 0.0001), respectively. The fold change in neutralizing antibody titers exhibited a weakly but significant negative correlation with age (r = −0.2961; P < 0.0001) (**Figure 5A**). Notably, participants aged 18 to 29 years showed the highest median (IQR) fold change (24[10-48]) in neutralizing antibody titers after vaccination (**Figure 5B**). Then, the fold changes declined in participants aged 30 to 39 years (16[6-32]), aged 40 to 49 years (12[4.998-32]), aged 50 to 59 years (12[6-24]) and aged 60 to 69 years (4[2-12]). However, there was a slight increase in the fold change in participants aged 70 to 79 years (6[2-15]). And, participants aged 80 years and above showed the lowest median (IQR) fold change (1.5[1-6]) in neutralizing antibody titers. Therefore, ZF2001 given as a booster vaccine induced a significantly lower degree of humoral immunogenicity in participants aged 60 years and above than those aged 18–59 years, and the immune response showed negative correlation with age. On Day 14 post-vaccination, there was no significant difference in neutralization titers among the three prime-boost interval groups, in both the younger and older age groups.

At baseline, the positive rates of neutralizing antibodies (titer ≥1:4) were nearly 50% in each group, except 18- to 59-year-olds in Group B, who had a rate of 70.8%. Additionally, there was no statistically significant difference in the antibody positive rates between 18–59 and 60-year-olds among Groups A–C (all *P* > 0.05) (**Table 2**). For participants aged 18–59 years, the neutralizing antibody positive rates were significantly different among the groups given the booster at different time intervals after the second dose of the inactivated vaccine (*P* = 0.002), but there was no significant difference for ≥60-year-old participants (*P* = 0.803) (**Table S2**). On Day 14, the antibody positive rates of participants aged 18–59 were above 98%, while those of subjects aged 60 and above were lower, at around 85%, within Groups A–C, and the differences were statistically significant (*P* = 0.009, *P* = 0.001, and *P* = 0.002). However, a comparison of the positive rates of neutralizing antibodies among the three interval groups showed no significant difference for participants aged both 18–59 and ≥60 years (*P* = 0.464 and *P* = 0.947, respectively). The seroconversion rates on Day 14 for Group A (seropositive/total) were 88.2% (90/102) and 65.0% (26/40); those for Group B were 90.0% (108/120) and 67.5% (27/40); and those for Group C were 90.8% (109/120) and 60.5% (23/38) for participants aged 18–59 and ≥60 years, respectively. The seroconversion rates differed significantly between 18–59 and 60-year-old participants in all three prime-boost interval groups (*P* =0.001, *P* = 0.001, and *P*< 0.001). However, a comparison of seroconversion rates among the three interval groups on Day 14 showed no significant difference for participants aged both 18–59 and ≥60 years (P = 0.856 and P = 0.836, respectively).

**Table 2:** Different groups with seropositivity rates and seroconversion rates at each timepoint.

Group	Seropositivity rates % (seropositive/total)						Seroconversion rates % (seroconversion/total)		
	Day 0	χ2	*P*	Day 14	χ2	*P*	Day 14	χ2	*P*
Group A Prime-boost 3-4 months interval									
Age 18-59 y	52.9 (54/102)	0.1	0.752	98.0 (100/102)	6.9	0.009*	88.2 (90/102)	10.4	0.001
Age ≥60 y	50.0 (20/40)			85.0 (34/40)			65.0 (26/40)		
Group B Prime-boost 5-6 months interval									
Age 18-59 y	70.8 (85/120)	2.4	0.119	100.0 (120/120)	11.6	0.001*	90.0 (108/120)	11.5	0.001
Age ≥60 y	57.5 (23/40)			87.5 (35/40)			67.5 (27/40)		
Group C Prime-boost 7-9 months interval									
Age 18-59 y	50.0 (60/120)	0.08	0.777	98.3 (118/120)	9.2	0.002*	90.8 (109/120)	19.3	<0.001
Age ≥60 y	52.6 (20/38)			84.2 (32/38)			60.5 (23/38)		

P-values result from a comparison between the two age sub-groups using two-sided chi-squared tests for categorical data. * using an adjusted chi-squared test

Seroconversion was defined as a change from a pre-immunization neutralizing antibody titer of<1:4 to a post-immunization one of 1:4 or a pre-immunization neutralizing antibody titer of 1:4 exhibiting a ≥4-fold increase.

## Discussion

In this study, we have shown that a third booster dose of recombinant protein subunit vaccine (ZF2001) safely provides a substantial increase in immune responses when administered 3–9 months after the second dose of CoronaVac in adults aged 18–59 and ≥60 years. Most adverse reactions were mild (grade 1), with the most common symptom being injection site pain, which is in accordance with previous findings (7, 20). There were no severe adverse reactions (grade 3) within 30 days of the booster.

After two doses of the inactivated vaccine (CoronaVac), neutralizing antibody titers at 3–9 months were close to negative for half of the participants, similar to the findings of another study, RHH-001, which detected titers 6 months after two doses of CoronaVac (21). These results show the antibody level attenuation that occurs after the administration of the two-dose CoronaVac. However, a study in Huashan hospital showed that, 4–8 months following the two-dose vaccination, participant’s neutralizing GMTs were still detectable (22), thus the findings were inconsistent with those of our study. The different study population and laboratory assays might account for this discrepancy. The study in Huashan hospital used plasma pseudovirus neutralization test (pVNT) to detect neutralizing titers, while we used CPE. More data are required from investigations of immune persistence after vaccination with two doses of CoronaVac before generalizing the results to larger populations from different countries and regions.

The use of ZF2001 as a third booster dose following the two-dose inactivated vaccine significantly recalled and increased immune responses by 3- to 16-fold compared with before the third dose. These findings indicate that the two-dose inactivated prime vaccine elicited durable humoral immunity that was successfully recalled by a third booster dose of the recombinant protein subunit vaccine (ZF2001) to provide protection against SARS-CoV-2. These individual datasets are important for policy makers to promote the benefits of heterologous third vaccinations with ZF2001 in China. However, the neutralizing antibody decay rate and long-term immune persistence still need to be evaluated in follow-up studies.

Our study found that the time interval between the third and second doses did not affect the immune response. In participants aged 18–59 and 60 years and above, there was no significant difference among the 3–9-month time interval groups after the second dose. A previous study found that antibody levels were higher among those vaccinated with the booster at longer intervals after the second vaccination dose (23). The authors reasoned this was because the maturation of memory B cells can take months after vaccination (24, 25). However, Zhang et al. found no significant difference in antibody responses among individuals given ZF2001 as a third dose after time intervals of 4–8 months, similar to the results of our research (22).

In this study, we demonstrated that the immune response to the booster vaccination was weaker in older adults than in younger adults, regardless of the interval of time that passed after the second dose of CoronaVac. After administration of ZF2001, GMTs in those 60 years and above were lower than those of 18–59 years (GMR2 = 0.41, 0.38, and 0.27). There was a significant decrease in positive rates and seroconversion rates on Day14 for those ≥60 years of age compared with those 18–59 years of age. Also, the immune response exhibited a weakly but significant negative correlation with age. The trend in the fold change in neutralizing antibody titers declined with age. This fact is also true for vaccines such as ChAdOx1 nCoV-19, as shown in previous studies (26), because of the age-associated decrease in immune function.

Our study had several limitations. Firstly, a homologous control group was not included. Homologous vaccination and heterologous vaccination could not be compared in this study, although we compared these with other studies. Secondly, the sample size was limited. More data are required to generalize the findings to larger populations and to optimize the timeframe for the third booster dose. Furthermore, neutralization testing against emerging VOC was not performed. SARS-CoV-2 variants continue to evolve, and the Omicron strain has become predominant (27). However, neutralizing antibody titers against the ancestral strain are considered important for protection against novel circulating SARS-CoV-2 variants. Lastly, the follow-up period was short, and the long-term safety profile of the third vaccination and the durability of the immune response following the booster are unclear. A follow-up of 6 months after the booster to determine the safety and immunogenicity of these regimens is ongoing.

One strength of our study is that it is the first to report on ZF2001 as a third-dose vaccination in an older age group in China. This study has provided vital data to help reduce the hesitancy to use a third dose of the ZF2001 COVID-19 vaccine in older adults. Additionally, this study provided a measure of pure vaccine-induced immunity unaffected by exogenous boosting by natural infection.

In conclusion, we found that the use of a recombinant protein subunit vaccine (ZF2001) booster against COVID-19 following a two-dose inactivated vaccine (CoronaVac) is safe and provides a strong immune response.

## Data availability statement

The original contributions presented in the study are included in the article/**Supplementary Material**. Further inquiries can be directed to the corresponding authors.

## Ethics statement

The studies involving human participants were reviewed and approved by Ethics Committee of Zhejiang Provincial Center for Disease Control and Prevention (2021-029-01). The participants provided their written informed consent to participate in this study.

## Author contributions

HL and YC contributed to study conception and design. CY, BC, and QZ recruited participants and conducted the study. QZ, XH, and BX provided technical and material support. YL and QH contributed to statistical analysis. YL and JY participated in the drafting the manuscript. HL revised the manuscript. All authors have read and agreed to the published version of the manuscript.

## Funding

This research was supported by the Key Program of Health Commission of Zhejiang Province/Science Foundation of National Health Commission under grant number WKJ-ZJ-2221; The Key Research and Development Program of Zhejiang Province, under grant number 2021C03200; Medical and Health Science and Technology Program of Zhejiang Province under grant number 2022KY713.

## Acknowledgments

We sincerely thank Anhui Zhifei Longcom Biopharmaceuticals for providing the ZF2001 vaccines and Chinese Center for Disease Control and Prevention for Live SARS-CoV-2 neutralization assay. We would like to express our sincere gratitude to all participants in this study.

## Conflict of interest

The authors declare that the research was conducted in the absence of any commercial or financial relationships that could be construed as a potential conflict of interest.

## Publisher’s note

All claims expressed in this article are solely those of the authors and do not necessarily represent those of their affiliated organizations, or those of the publisher, the editors and the reviewers. Any product that may be evaluated in this article, or claim that may be made by its manufacturer, is not guaranteed or endorsed by the publisher.
