# Supplementary material for: Safety and immunogenicity of heterologous recombinant protein subunit vaccine (ZF2001) booster against COVID-19 at 3–9-month intervals following two-dose inactivated vaccine (CoronaVac)
Source: Front Immunol. 2022 Nov 8;13:1017590. doi: 10.3389/fimmu.2022.1017590 (PMC9679005; doi:10.3389/fimmu.2022.1017590)
Supplement: Supplementary file 1 [file DataSheet_1.docx]

Supplementary Material

# Supplementary Methods

Inclusion and Exclusion Criteria

Inclusion criteria:

1. Participants aged 18 years and above who received priming vaccination with two doses of inactivated vaccine (CoronaVac) within 3 to 9 months prior to the study.

2. Participants who voluntarily took part in the study and signed the informed consent, and were also willing to comply with the study procedures.

3. Female participants of childbearing potential who agreed to use effective contraception from the start of the study to 2 months after the booster vaccination.

Exclusion criteria:

1. Axillary temperature of ≥37.5°C within 72 hours prior to Day 0 of vaccination or ≥37.3°C on the day of enrollment.

2. Uncontrollable hypertension (diastolic blood pressure ≥100 mmHg and/or systolic blood pressure ≥160 mmHg).

3. A history of infection with SARS-CoV-2 or SARS-CoV-2 infection confirmed by positive reverse-transcription-polymerase-chain reaction (RT-PCR) assay.

4. Presence of fever, cough, runny nose, sore throat, diarrhea, dyspnea, or tachypnea within 3 days before screening visit.

5. A history of severe allergy (such as angioneurotic oedema or allergic shock) to any

vaccination.

6. Were administered subunit vaccines or inactivated vaccines within 14 days or live attenuated vaccines within 28 days before the booster vaccination.

7. Thrombocytopenia, any coagulation disorder, or recipient of anticoagulant therapy.

8. A history or family history of mental illness or serious central nervous system diseases (such as epilepsy, transverse myelitis, Guillain Barre syndrome, demyelinating disease, encephalopathy).

9. Serious chronic diseases, such as congenital heart disease, severe liver and kidney disease, or severe diabetes (with complications), that may interfere with the conduct or completion of the study.

10. Cancer patients (except basal cell carcinoma) who had been off treatment for less than 6 months.

11. Other acute diseases or chronic diseases with acute exacerbation.

12. Known history of cancer or solid organ transplant.

13. Known immunosuppressive or immunodeficient state, including confirmed HIV

infection, and history of receiving systemic immunosuppressants within 3 months prior to the day of screening.

14. Participating in other COVID-19-related clinical trials.

15. Pregnant women (including women of childbearing age who had a positive urine pregnancy test).

# Supplementary Tables

Table S1. Adverse reactions within 30 days after booster vaccination

| Adverse reactions within 0–30 days | Group A | | Group B | | Group C | | Overall | |
| --- | --- | --- | --- | --- | --- | --- | --- | --- |
|  | (N=160) | | (N=160) | | (N=160) | | (N=480) | |
|  | n | (%) | n | (%) | n | (%) | n | (%) |
| All adverse reactions | 13 | 8.13 | 9 | 5.63 | 6 | 3.75 | 28 | 5.83 |
| Grade 1 | 12 | 7.5 | 5 | 3.13 | 6 | 3.75 | 23 | 4.79 |
| Grade 2 | 2 | 1.25 | 4 | 2.5 | 0 | 0 | 6 | 1.25 |
| Grade 3 | 0 | 0 | 0 | 0 | 0 | 0 | 0 | 0 |
| All solicited adverse reactions | 13 | 8.13 | 9 | 5.63 | 5 | 3.13 | 27 | 5.63 |
| Grade 1 | 12 | 7.5 | 5 | 3.13 | 5 | 3.13 | 22 | 4.58 |
| Grade 2 | 2 | 1.25 | 4 | 2.5 | 0 | 0 | 6 | 1.25 |
| Grade 3 | 0 | 0 | 0 | 0 | 0 | 0 | 0 | 0 |
| Solicited injection site adverse reactions | 5 | 3.13 | 5 | 3.13 | 3 | 1.88 | 13 | 2.71 |
| Grade 1 | 5 | 3.13 | 3 | 1.88 | 3 | 1.88 | 11 | 2.29 |
| Grade 2 | 0 | 0 | 2 | 1.25 | 0 | 0 | 2 | 0.42 |
| Grade 3 | 0 | 0 | 0 | 0 | 0 | 0 | 0 | 0 |
| Pain | 4 | 2.5 | 5 | 3.13 | 2 | 1.25 | 11 | 2.29 |
| Grade 1 | 4 | 2.5 | 3 | 1.88 | 2 | 1.25 | 9 | 1.88 |
| Grade 2 | 0 | 0 | 2 | 1.25 | 0 | 0 | 2 | 0.42 |
| Grade 3 | 0 | 0 | 0 | 0 | 0 | 0 | 0 | 0 |
| Redness | 0 | 0 | 0 | 0 | 0 | 0 | 0 | 0 |
| Grade 1 | 0 | 0 | 0 | 0 | 0 | 0 | 0 | 0 |
| Grade 2 | 0 | 0 | 0 | 0 | 0 | 0 | 0 | 0 |
| Grade 3 | 0 | 0 | 0 | 0 | 0 | 0 | 0 | 0 |
| Swelling | 0 | 0 | 0 | 0 | 0 | 0 | 0 | 0 |
| Grade 1 | 0 | 0 | 0 | 0 | 0 | 0 | 0 | 0 |
| Grade 2 | 0 | 0 | 0 | 0 | 0 | 0 | 0 | 0 |
| Grade 3 | 0 | 0 | 0 | 0 | 0 | 0 | 0 | 0 |
| Induration | 0 | 0 | 0 | 0 | 0 | 0 | 0 | 0 |
| Grade 1 | 0 | 0 | 0 | 0 | 0 | 0 | 0 | 0 |
| Grade 2 | 0 | 0 | 0 | 0 | 0 | 0 | 0 | 0 |
| Grade 3 | 0 | 0 | 0 | 0 | 0 | 0 | 0 | 0 |
| Rash | 0 | 0 | 0 | 0 | 0 | 0 | 0 | 0 |
| Grade 1 | 0 | 0 | 0 | 0 | 0 | 0 | 0 | 0 |
| Grade 2 | 0 | 0 | 0 | 0 | 0 | 0 | 0 | 0 |
| Grade 3 | 0 | 0 | 0 | 0 | 0 | 0 | 0 | 0 |
| Pruitus | 3 | 1.88 | 0 | 0 | 1 | 0.63 | 4 | 0.83 |
| Grade 1 | 3 | 1.88 | 0 | 0 | 1 | 0.63 | 4 | 0.83 |
| Grade 2 | 0 | 0 | 0 | 0 | 0 | 0 | 0 | 0 |
| Grade 3 | 0 | 0 | 0 | 0 | 0 | 0 | 0 | 0 |
| Solicited systemic adverse reactions | 9 | 5.63 | 4 | 2.5 | 2 | 1.25 | 15 | 3.13 |
| Grade 1 | 8 | 5 | 2 | 1.25 | 2 | 1.25 | 12 | 2.5 |
| Grade 2 | 2 | 1.25 | 2 | 1.25 | 0 | 0 | 4 | 0.83 |
| Grade 3 | 0 | 0 | 0 | 0 | 0 | 0 | 0 | 0 |
| Fever | 1 | 0.63 | 0 | 0 | 1 | 0.63 | 2 | 0.42 |
| Grade 1 | 1 | 0.63 | 0 | 0 | 1 | 0.63 | 2 | 0.42 |
| Grade 2 | 0 | 0 | 0 | 0 | 0 | 0 | 0 | 0 |
| Grade 3 | 0 | 0 | 0 | 0 | 0 | 0 | 0 | 0 |
| Headache | 5 | 3.13 | 2 | 1.25 | 1 | 0.63 | 8 | 1.67 |
| Grade 1 | 4 | 2.5 | 1 | 0.63 | 1 | 0.63 | 6 | 1.25 |
| Grade 2 | 1 | 0.63 | 1 | 0.63 | 0 | 0 | 2 | 0.42 |
| Grade 3 | 0 | 0 | 0 | 0 | 0 | 0 | 0 | 0 |
| Fatigue | 3 | 1.88 | 1 | 0.63 | 0 | 0 | 4 | 0.83 |
| Grade 1 | 3 | 1.88 | 0 | 0 | 0 | 0 | 3 | 0.63 |
| Grade 2 | 0 | 0 | 1 | 0.63 | 0 | 0 | 1 | 0.21 |
| Grade 3 | 0 | 0 | 0 | 0 | 0 | 0 | 0 | 0 |
| Diarrhea | 1 | 0.63 | 1 | 0.63 | 0 | 0 | 2 | 0.42 |
| Grade 1 | 1 | 0.63 | 1 | 0.63 | 0 | 0 | 2 | 0.42 |
| Grade 2 | 0 | 0 | 0 | 0 | 0 | 0 | 0 | 0 |
| Grade 3 | 0 | 0 | 0 | 0 | 0 | 0 | 0 | 0 |
| Nausea | 2 | 1.25 | 0 | 0 | 0 | 0 | 2 | 0.42 |
| Grade 1 | 2 | 1.25 | 0 | 0 | 0 | 0 | 2 | 0.42 |
| Grade 2 | 0 | 0 | 0 | 0 | 0 | 0 | 0 | 0 |
| Grade 3 | 0 | 0 | 0 | 0 | 0 | 0 | 0 | 0 |
| Vomiting | 0 | 0 | 0 | 0 | 0 | 0 | 0 | 0 |
| Grade 1 | 0 | 0 | 0 | 0 | 0 | 0 | 0 | 0 |
| Grade 2 | 0 | 0 | 0 | 0 | 0 | 0 | 0 | 0 |
| Grade 3 | 0 | 0 | 0 | 0 | 0 | 0 | 0 | 0 |
| Muscle ache | 1 | 0.63 | 0 | 0 | 0 | 0 | 1 | 0.21 |
| Grade 1 | 1 | 0.63 | 0 | 0 | 0 | 0 | 1 | 0.21 |
| Grade 2 | 0 | 0 | 0 | 0 | 0 | 0 | 0 | 0 |
| Grade 3 | 0 | 0 | 0 | 0 | 0 | 0 | 0 | 0 |
| Acute allergic reaction | 1 | 0.63 | 0 | 0 | 0 | 0 | 1 | 0.21 |
| Grade 1 | 0 | 0 | 0 | 0 | 0 | 0 | 0 | 0 |
| Grade 2 | 1 | 0.63 | 0 | 0 | 0 | 0 | 1 | 0.21 |
| Grade 3 | 0 | 0 | 0 | 0 | 0 | 0 | 0 | 0 |
| Cough | 1 | 0.63 | 1 | 0.63 | 0 | 0 | 2 | 0.42 |
| Grade 1 | 1 | 0.63 | 0 | 0 | 0 | 0 | 1 | 0.21 |
| Grade 2 | 0 | 0 | 1 | 0.63 | 0 | 0 | 1 | 0.21 |
| Grade 3 | 0 | 0 | 0 | 0 | 0 | 0 | 0 | 0 |
| All unsolicited adverse reactions | 0 | 0 | 0 | 0 | 1 | 0.63 | 1 | 0.21 |
| Grade 1 | 0 | 0 | 0 | 0 | 1 | 0.63 | 1 | 0.21 |
| Grade 2 | 0 | 0 | 0 | 0 | 0 | 0 | 0 | 0 |
| Grade 3 | 0 | 0 | 0 | 0 | 0 | 0 | 0 | 0 |
| Dizziness | 0 | 0 | 0 | 0 | 1 | 0.63 | 1 | 0.21 |
| Grade 1 | 0 | 0 | 0 | 0 | 1 | 0.63 | 1 | 0.21 |
| Grade 2 | 0 | 0 | 0 | 0 | 0 | 0 | 0 | 0 |
| Grade 3 | 0 | 0 | 0 | 0 | 0 | 0 | 0 | 0 |

Data are n and proportion of participants (%). Adverse reactions and reactions were graded according to the scale issued by the China State Food and Drug Administration. Grade 1 is mild, grade 2 is moderate, and grade 3 is severe.

Table S2: Different groups with seropositivity rates and seroconversion rates at each timepoint

| Group | Seropositivity rates % (seropositive/total) | | | | | | Seroconversion rates % (seroconversion /total) | | |
| --- | --- | --- | --- | --- | --- | --- | --- | --- | --- |
|  | Day 0 | χ2 | P | Day 14 | χ2 | P | Day 14 | χ2 | P |
| Age 18-59 y |  | 12.3 | 0.002 |  | 2.3** | 0.464 |  | 0.4 | 0.856 |
| Group A | 52.9 (54/102) |  |  | 98.0 (100/102) |  |  | 88.2 (90/102) |  |  |
| Group B | 70.8 (85/120) |  |  | 100.0 (120/120) |  |  | 90.0 (108/120) |  |  |
| Group C | 50.0 (60/120) |  |  | 98.3 (118/120) |  |  | 90.8 (109/120) |  |  |
| Age ≥60 y |  | 0.5 | 0.803 |  | 0.2 | 0.947 |  | 0.4 | 0.836 |
| Group A | 50.0 (20/40) |  |  | 85.0 (34/40) |  |  | 65.0 (26/40) |  |  |
| Group B | 57.5 (23/40) |  |  | 87.5 (35/40) |  |  | 67.5 (27/40) |  |  |
| Group C | 52.6 (20/38) |  |  | 84.2 (32/38) |  |  | 60.5 (23/38) |  |  |

P-values result from a comparison between the three groups using two-sided chi-squared tests for categorical data. ** using Fisher’s exact test

Seroconversion was defined as a change from a pre-immunization neutralizing antibody titer of <1:4 to a post-immunization one of 1:4 or a pre-immunization neutralizing antibody titer of 1:4 exhibiting a ≥4-fold increase.
